# Editorial: Stomatal conductance in a changing climate: mechanistic insights and ecosystem feedbacks

**DOI:** 10.3389/fpls.2026.1991994

**Published:** 2026-09-18

**Authors:** Yun Bai, Sha Zhang, Tian Hu, Ren Wang, Jingwen Wang

**Affiliations:** 1 School of Geographical Sciences, Hebei Normal University, Shijiazhuang, China; 2 Luxembourg Institute of Science and Technology (LIST), Esch-sur-Alzette, Luxembourg; 3 School of Atmospheric Sciences, Nanjing University of Information Science and Technology, Nanjing, China; 4 Shenzhen Institutes of Advanced Technology, Chinese Academy of Sciences, Shenzhen, China

**Keywords:** carbon-water trade-off, climate change drivers, ecosystem services and feedbacks, multi-scale integration (leaf-to-ecosystem), plant hydraulic traits, stomatal conductance, stomatal optimization models, water-use efficiency

Stomatal conductance (*g*_s_) is a key physiological trait that couples plant carbon uptake with water loss, and it is a central determinant of the terrestrial carbon and water cycles under a changing climate. Rising atmospheric CO_2_, intensifying drought stress, more frequent heatwaves and elevated vapor pressure deficit (VPD) are jointly altering stomatal behaviour and, thereby modifying ecosystem water-use efficiency, carbon assimilation, and vegetation–atmosphere feedbacks. Yet the response of g_s_ to these drivers remains difficult to predict, because stomatal regulation is governed by the interplay of anatomical, physiological, biochemical and hydraulic traits that act across multiple spatial and temporal scales, from the sub-cellular level to the canopy and beyond. The six contributions collected in this Research Topic combine controlled experiments, field observations, genetic analyses, mechanistic modelling and ecosystem-scale assessments to advance our understanding of these multi-scale interactions. Together, they emphasize that robustly projecting g_s_ under climate change requires the integration of leaf-level physiology with hydraulic, genetic and ecosystem perspectives. This multi-scale structure is illustrated in **Figure 1**, which links the principal climate drivers to the central g_s_ trade-off and situates each contribution along a leaf-to-ecosystem axis.

**Figure 1:**
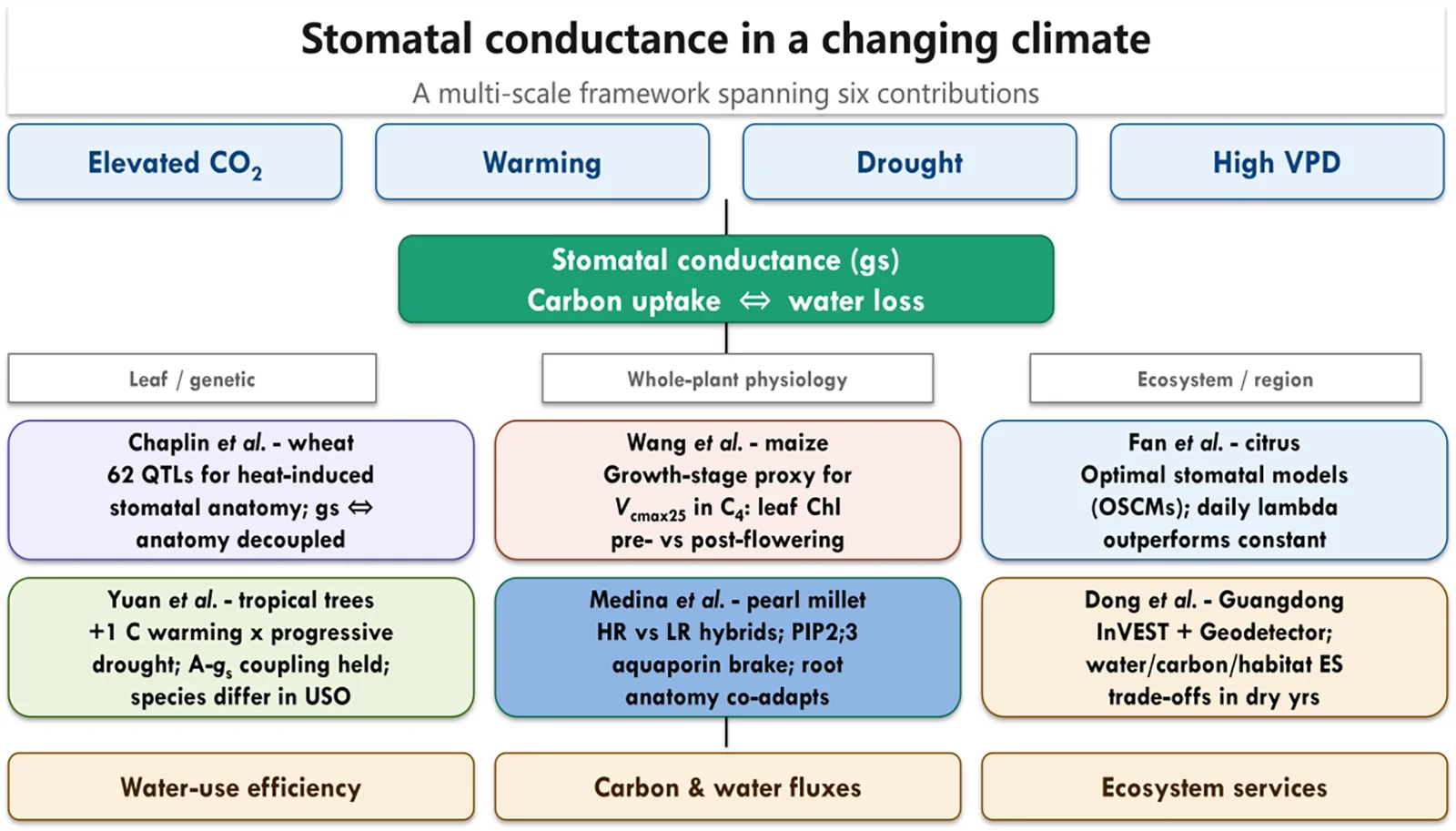
Conceptual framework of the Research Topic. Climate drivers (top) reshape stomatal conductance (*g*_s_); the six contributions are arranged along a leaf-to-ecosystem axis; outputs flow toward water-use efficiency, carbon and water fluxes, and ecosystem services.

The collection spans a broad taxonomic and methodological range that reflects the multi-scale scope of the topic. Two studies use field-based approaches to examine the heritable basis of stomatal acclimation. Chaplin et al. characterized a panel of 200 wheat genotypes across two growing seasons and contrasting sowing times to assess how heat stress affects stomatal anatomy, conductance and operating efficiency. They identified 62 quantitative trait loci, including recurring loci on chromosomes 6B, 1A, 2A and 7A, and showed that heat stress uncouples structural capacity from physiological performance, providing a basis for breeding heat-tolerant wheat ideotypes. Medina et al. compared pearl millet hybrids selected in high- versus low-rainfall zones to evaluate the influence of breeding history on root water uptake and aquaporin regulation. Low-rainfall hybrids showed greater root and canopy development, supported by extensive metaxylem proliferation and sustained aquaporin PIP2;3 expression, which maintained radial water transport under high transpirational demand, whereas high-rainfall hybrids downregulated PIP2;3, reducing root hydraulic conductance and conserving water. These two studies show that adaptation to a given hydroclimate can be achieved through contrasting anatomical and hydraulic strategies, with implications for breeding crops for projected future climates.

A second theme addresses the covariation of stomatal behaviour with photosynthesis and hydraulic transport under compound stress. Yuan et al. exposed seedlings of three dominant subtropical tree species to simultaneous warming (+1 °C) and progressive drought in open-top chambers, and observed that the coupling between stomatal conductance and net assimilation remained stable across species despite substantial drought-induced reductions in photosynthesis. Leaf CO_2_ supply was the dominant limitation, with reduced diffusion through both stomatal and mesophyll pathways accounting for most of the decline and no clear evidence of photochemical damage. Species nevertheless differed in stomatal strategy: *Castanopsis carlesii* showed a higher slope parameter of the unified stomatal optimization model, indicative of a more profligate behaviour, whereas *Schima superba* and *Cunninghamia lanceolata* were more conservative. These results argue against a single generic parameterization of land-surface models and recommend species-specific stomatal parameters for simulating subtropical forest carbon and water fluxes. Wang et al. examined whether the chlorophyll-based proxy for photosynthetic capacity developed for C_3_ crops transfers to the C_4_ crop maize. Weekly measurements over the full growing season revealed that leaf chlorophyll content was the strongest predictor of the maximum carboxylation rate (*V*_cmax25_) before flowering (R^2^ = 0.81), but its predictive power declined afterwards, indicating that growth-stage information is necessary when using leaf optical properties to estimate photosynthetic capacity in C_4_ canopies.

Modelling the *g*_s_ response to environmental variability is addressed by Fan et al., who used a full phenological cycle of citrus gas-exchange measurements to parameterize a family of optimal stomatal conductance models (OSCMs). Model accuracy depended strongly on both the assumed biochemical limitation and the temporal resolution of the marginal water-cost parameter *λ*; the RuBP-regeneration-limited, daily-*λ* form (OSCvjd) performed best (R^2^ = 0.73). These models reproduced intercellular CO_2_ concentration and photosynthesis reasonably well and required only routine meteorological variables, making them applicable to orchard water management in subtropical regions where drought stress is increasing. Together with the wheat and millet studies, this work illustrates that optimality-based frameworks—most notably the Cowan–Farquhar theory and its modern descendants such as the unified stomatal optimization model—remain useful for interpreting the carbon-for-water trade-off across crops and climates, provided that their parameters are tuned to the species, stress regime and temporal scale of interest.

Finally, Dong et al. extend the analysis from leaf and plant scales to the regional ecosystem scale. Applying the InVEST model, a coupling-coordination model and Geodetector to Guangdong Province from 2000 and 2020, they show that the trade-offs and synergies among water conservation, habitat quality, and net primary production differ among urban, cultivated, and natural ecosystems and become more pronounced in dry years. Their finding—that long-term water-conservation projects in western Zhaoqing, Yunfu, southeastern Meizhou and the Chaoshan Plain may not effectively improve the overall bundle of ecosystem services during droughts— indicates that regional hydrological interventions cannot substitute for a process-based understanding of the vegetation, including stomatal regulation, that govern carbon and water exchange.

Taken together, the six contributions in this Research Topic support three cross-cutting conclusions. First, the genetic and anatomical architecture of stomata is as important as the physiological response: heritable variation in stomatal density, size and aquaporin regulation provides a tractable route to improving climate resilience, and intraspecific diversity remains an underused resource in many crops and natural ecosystems. Second, stomatal behaviour under climate change is a multi-stress, multi-scale phenomenon: warming, drought, VPD and ontogeny interact in ways that no single experiment or model can capture, and analyses combining controlled multi-factor designs with hierarchical modelling are particularly informative. Third, linking leaf-level physiology to ecosystem-level outcomes requires more than scaling up gas-exchange equations; it requires integrating trait-based, optimality-based and process-based frameworks with spatially explicit remote-sensing and ecosystem-service analyses.

We hope this collection will serve both as a synthesis of the current state of the field and as an agenda for future work—particularly for studies that use emerging remote-sensing products, eddy-covariance networks, and genomic resources to constrain the *g*_s_ response across spatial and temporal scales. As climate change accelerates, such integration becomes increasingly urgent, and we thank all the authors who contributed to this Research Topic for sharing their work.

